# Application of concentrated deep sea water inhibits the development of atopic dermatitis-like skin lesions in NC/Nga mice

**DOI:** 10.1186/1472-6882-12-108

**Published:** 2012-07-26

**Authors:** Jong-Phil Bak, Yong-Min Kim, Jeonghyun Son, Chang-Ju Kim, Ee-Hwa Kim

**Affiliations:** 1 The Clinical Trial Center for Bio-Industry, Semyung University, Jecheon 309-711, South Korea; 2 Department of Physiology, College of Medicine, Kyung Hee University, Seoul 130-701, South Korea

## Abstract

**Background:**

Mineral water from deep-sea bedrock, formed over thousands of years, is rich in minerals such as Ca, Mg, Na, K, Fe and others. Our present study was to investigate the preventive effects of natural deep-sea water on developing atopic dermatitis (AD).

**Methods:**

We elicited AD by application of DNCB (2,4-dinitro-chlorobezene) in Nc/Nga mouse dorsal skin. Deep Sea water (DSW) was filtered and concentrated by a nanofiltration process and reverse osmosis. We applied concentrated DSW (CDSW) to lesions five times per week for six weeks, followed by evaluation. 1% pimecrolimus ointment was used as positive control. The severity of skin lesions was assessed macroscopically and histologically. Levels of inflammatory mediators and cytokines in the serum were detected by Enzyme-linked immunosorbent assay (ELISA) and the levels of CD4^+^ and CD8^+^ spleen lymphocytes were determined by flow cytometry analysis.

**Results:**

DNCB-treated mice showed atopic dermatitis-like skin lesions. Treatment of mice with CDSW reduced the severity of symptoms in the skin lesions, including edema, erythema, dryness, itching, and transepidermal water loss (TEWL). Histological analyses demonstrated that epidermal thickness and infiltration of inflammatory cells were decreased after CDSW treatment. Given these interesting observations, we further evaluated the effect of CDSW on immune responses in this AD model. Treatment AD mice with CDSW inhibited up-regulation of IgE, histamine, and pro-inflammatory cytokines in the serum. Also, the CD4^+^/CD8^+^ ratio in spleen lymphocyte was down-regulated after treatment with CDSW. Finally, cytokines, especially IL-4 and IL-10 which are important for Th2 cell development, were reduced.

**Conclusions:**

Our data suggests that topical application of CDSW could be useful in preventing the development of atopic dermatitis.

## Background

Mineral water from deep-sea bedrock is an attractive prospect because it is rich in nutrients and minerals such as Ca, Mg, Na, Zn, K, Fe, HCO_3_, Cl, SO_4_, NO_3_, etc. (Table 1) [1]. Iron, in particular, is abundant in the deep-sea water (DSW). Therefore, it seems to have an effect on the prevention or treatment of anemia [2,3].

**Table 1:** The levels of elements in pre-DSW and CDSW

**Elements**	**Pre DSW (mg/L)**	**CDSW (mg/L)**
Na	6300	4070
Cl^-^	18200	309400
Ca	2950	102100
Mg	714	34800
K	28.1	1950
Si	9.07	2.34
Mn	2.16	35.3
Zn	0.08	1.05
Fe	0.06	0.12
Cu	0.02	0.48
B	1.38	20.7

Na: sodium; Cl: chlorine; Ca: calcium; Mg: magnesium; K: potassium; Si: silicon; Mn: manganese; Zn: zinc; Fe: iron Cu: copper; B: boron.

Atopic dermatitis (AD) is a chronic inflammatory skin disease with symptoms of dryness and itchiness, in which relapse is common [4,5]. The prevalence of AD has increased 2–3 fold during the past three decades in industrialized societies [5-9]. In the past a few years, it was reported that pathogenesis of AD was related to a complex interrelation of immunological, psychological, environmental and genetic factors [10]. AD skin lesions are characterized by the presence of infiltrated inflammatory cells such as eosinophils, monocytes, macrophages, mast cells or T lymphocytes [11-14]. Several studies have been performed to investigate cytokines, and reports show that the generation of AD is elicited by a pathological defect, such as cytokines from T-helper (Th) cells [15].

Th cells can be divided into two distinct groups in accordance with their cytokines; Th1 Th2 lymphocytes. In AD patients’ blood, IL-4 and IL-5 concentration from Th2 cells was increased and IL-4, IL-5 and IL-13 mRNA expression was significantly up-regulated in atopic skin lesions [16,17]. However, recent studies reported that Th1 response also plays an important role in AD disease [12]. In almost all chronic AD patients, up-regulation of the expression IFN-γ mRNA was also observed in atopic skin lesions, whereas only a few chronic AD patients had increases in the expression of IL-4 mRNA [18,19]. Therefore, both Th1 and Th2 response contribute to the initiation and maintenance of AD.

Treatment for AD has been studied using such drugs based on steroids, anti-histaminic agents, and immunosuppressants [20]. However, there are many side effects and limited therapeutic effects with long-term use [21]. Recently, it was reported that the application of minerals has a therapeutic effect on the skin diseases [22-25]. Other studies reported that drinking mineral water or exposure to mineral water at the spa affects the immune system [1,26]. The effect of deep-sea water (DSW) on atopic dermatitis is poorly understood. Therefore, we wanted to demonstrate whether application of DSW to atopic skin lesions has any beneficial effects for preventing atopic dermatitis in a mouse model for AD [27].

## Methods

### Chemicals and reagents

Natural deep-sea water (DSW) and concentrated DSW (CDSW) were supplied by Jin Hae Resource Development Inc. (Inchon, South Korea). The elemental composition of DSW and CDSW were determined by inductively coupled plasma mass spectrometry (ICP-MS). The mouse IgE ELISA assay kit was from Koma Biotech (Seoul, Korea), the mouse Th1/Th2 ELISA assay kit was from eBioscience (San Diego, USA), and the IL-1β, IL-6 and TNF-α ELISA assay kits were from R&D Systems (Minneapolis, MN, USA). The CD4 (GK1.5) Alexa Fluor 488 and CD8 (2.43) PE antibodies were from Santa Cruz Biotechnology (USA). Elidel (Novartis) is a 1% pimecrolimus topical solution and is an immunomodulating agent used for treating eczema [28]. All other reagents were from Sigma-Aldrich (St. Louis, MO, USA).

### Desalted and concentrated DSW

Deep-sea water (DSW) was selectively filtered for sodium chloride by nanofiltration and reverse osmosis. Desalinization was performed by pre-processing with Hytrex Cartridge Filter (Hytrex, USA), nano-filtration by SU-610 membrane (Toray, Japan), and reverse osmosis by SU-810 membrane (Toray, Japan), respectively (Additional file 1: Figure S1). Concentrated DSW (CDSW) was nano-filtered with SU-610 and evaporated (Additional file 2: Figure S2). The elemental composition of DSW (Patent Number: KR 10–0663084) and CDSW (Patent Number: KR 10–0670474) are shown in Table 1, and were confirmed by Korea Testing & Research Institute (KTR).

### Animals

Six-week old male NC/Nga mice were from SLC Inc. (Shizuoka, Japan). All experimental protocols were reviewed and approved by the Animal Care Committee at Semyung University (IACUC, protocol number: SMECAE 10-10-01). The mice were house in a conventional laboratory conditions with filtered air and consistent ambient temperature (24 ± 2°C), humidity (55 ± 10%) and light (12 h light/dark cycle). The mice were fed a non-purified pellet diet and tap water *ad libitum*. Mice were divided into five groups (n = 10 per group) as follows: A, normal control; B, negative control (DNCB); C, positive control (DNCB + 1% pimecrolimus); D, 2% CDSW treatment (DNCB + 2% CDSW); and E, 10% CDSW treatment (DNCB + 10% CDSW).

### DNCB-induced dermatitis

The experimental schedule for the preparation of AD-like skin lesions in NC/Nga mice is summarized in Additional file 3: Figure S3. The hair of dorsal skin of mice was shaved under Zoletil 50 (50 mg/kg) and xylazine (10 mg/kg) anaesthesia with a hair clipper 1 day before initial sensitization. On day 0, these mice were sensitized by applying 200 μL of a solution containing 1% DNCB in an acetone and olive oil mixture (3:1) to cause AD-like skin lesions. After initial challenge, 0.4% DNCB solution was repeatedly applied to the same area of the skin for 12 times at 3 day intervals. 200 μL of test samples (DW, 2% CDSW, 10% CDSW, 1% pimecrolimus) were applied to the dorsal skin every day (p.m. 2:00 ~ 3:00).

### The clinical skin severity

The clinical skin severity was evaluated once per week (a.m. 10:00 ~ 11:00) by the following scoring procedure; 0 (none), 1 (mild), 2 (moderate), 3 (severe) for each of the five indications and symptoms including itching, erythema, edema, excoriation/erosion, and scaling/dryness [28]. Itch was assessed by their behavior in 1 hour, and scaling/dryness by content of moisture in the epidermis using Corneometer CM825 (Courage and Khazaka Electronic Co., Germany). Erythema, edema, and excoriation/erosion were evaluated macroscopically.

### Measurement of transepidermal water loss on the dorsal skin

The hair on the dorsal skin of each mouse was shaved using an hair clipper under Zoletil 50 (50 mg/kg) and xylazine (10 mg/kg) anesthesia and TEWL was measured using a Tewameter TM300 (Courage and Khazaka Electronic Co., Germany) at 0, 1, 2, 3, 4, 5 and 6 weeks. The values were recorded when stabilized approximately 10 s after the probe had been placed on the skin.

### Histological analysis

Skin tissue was isolated from each group of mice and fixed with 10% 50 mM PFA in phosphate buffer (pH 7.0) for 24 hours at 4°C. Eight micrometer-thick sections of dorsal skin were stained with hematoxylin and eosin (H&E) and observed by optical microscopy (Evos Xl, USA). The sections were examined for the presence and degree of incrustation, thickness of the epidermis, epidermal necrosis, bleeding, hyperkeratosis and inflammation. Epidermal thickness was measured by Micron (EVOS, v2.0) a digital imaging software.

### Measurement of blood parameters

On the sixth week, the mice were sacrificed, blood was collected from the heart, and the spleen was dissected. The total IgE and histamine levels in the serum were measured by ELISA. Levels of inflammatory cytokines (IL-1β, IL-6 and TNF-α)and Th cell development cytokines (IL-2, IL-4, IL-10 and IFN-γ) in the serum also were determined by ELISA. ELISA was performed according to the manufacturer’s instruction and quantitation was done with SPECTRAMAX190 (Molecular Devices, USA).

### Preparation of spleen lymphocytes

To analyze spleen lymphocytes, the spleens were cut into pieces with a scissors in cold phosphate-buffered saline (PBS) and homogenized with a glass homogenizer. Homogenized spleen was passed through strainer (BD, USA). Spleen lymphocytes were isolated with Histopaque-1077 Hybri-Max (Sigma, USA) and centrifuged at 400 g for twenty minutes. Lymphocytes were washed three times with PBS. 10% fetal bovine serum (FBS)/phosphate-buffer saline (PBS) was added to the spleen lymphocytes suspended at 1 × 10^6^ cells/ml with 5ml of either CD4-Alexa Fluor 488 or CD8-PE monoclonal antibodies, and incubated at 4°C for thirty minutes. The lymphocytes were rinsed five times with PBS containing 10% FBS and centrifuged at 1,200 rpm for five minutes. The stained lymphocytes were fixed by 2% paraformaldehyde and counted by FACSCalibur™ flow cytometry (BD Bioscience, USA). Each analysis, including the control samples, was based on at least 1 x 10^4^ events exclusive of dead cells, and gating on the basis of forward angle light scatter eliminated residual erythrocytes [29].

### Statistical analysis

Results are presented as mean ± standard deviation (SD). Data were analysed by one-way analysis of variance (ANOVA) followed by Tukey’s test or Student’s *t*-test (*P* < 0.05).

## Results

### Effect of CDSW on atopic skin lesions

**Figure 1:**
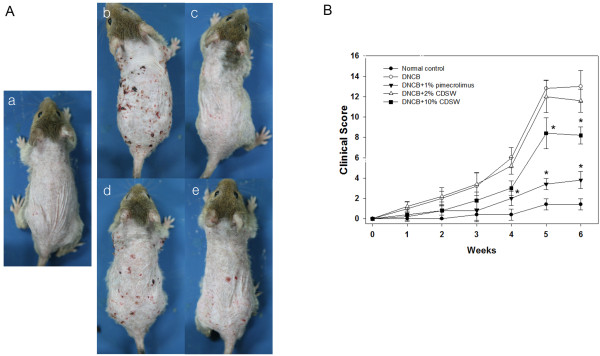
**Effect of CDSW on development of AD-like skin lesions in DNCB-treated NC/Nga mice.A**) The dorsal skin of mice were shaved and were sensitized by DNCB; a. normal control, b. Negative control (DNCB), c. Positive control (DNCB + 1% pimecrolimus), d. 2% CDSW-treated (DNCB + 2% CDSW) e. 10% CDSW-treated (DNCB + 10% CDSW); (n = 10). The skin lesions recovered and **B**) the clinical score of DNCB-treated mice was suppressed by the application of 10% CDSW (*p < 0.05).

**Figure 2:**
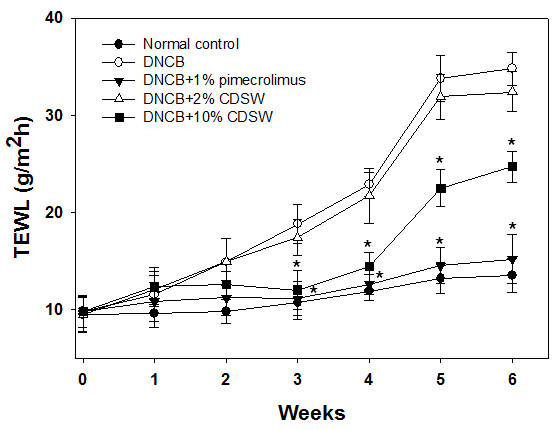
**The effect of CDSW on TEWL in DNCB-treated NC/Nga mice.** The TEWL was measured every week for 6 weeks after sensitization using Tewameter. Measurements were recorded when TEWL reading were stabilized at 10 s after the probe was placed on the skin. Data are presented as means ± SE (n = 10, *p < 0.05).

We first examined whether DSW has any preventive effect on atopic dermatitis. 1% pimecrolimus cream was used as atopic dermatitis medicine [30] and by the treatment of pimecrolimus, AD was prevented development significantly as observed by histological analysis (Figure 1A). DSW showed high salt concentration and maybe damage to skin lesions. Therefore, we used concentrated deep sea water (CDSW) that was desalted (Table 1). Interestingly, the CDSW-treated mice recovered from atopic symptoms in a concentration-dependent manner (Figure 1A). To verify the effect of CDSW treatment on AD skin lesions, we observed and scored skin symptoms including itching, erythema, edema, excoriation/erosion and scaling/dryness for six weeks (Figure 1B) [28]. After six weeks, the clinical score of the DNCB-treated group increased significantly, whereas the 1% pimecrolimus group was interfered with development of AD. The CDSW-treated group also inhibited increase of the clinical score, with the 10% CDSW-treated mice significantly. The TEWL of the DNCB-treated group was increased as time goes on. The 1% pimecrolimus- and 10% CDSW-treated groups significantly inhibited the increase of TEWL compared with the DNCB-treated group from three weeks (Figure 2).

### Recovery of skin tissue in atopic lesions by treatment of CDSW

**Figure 3:**
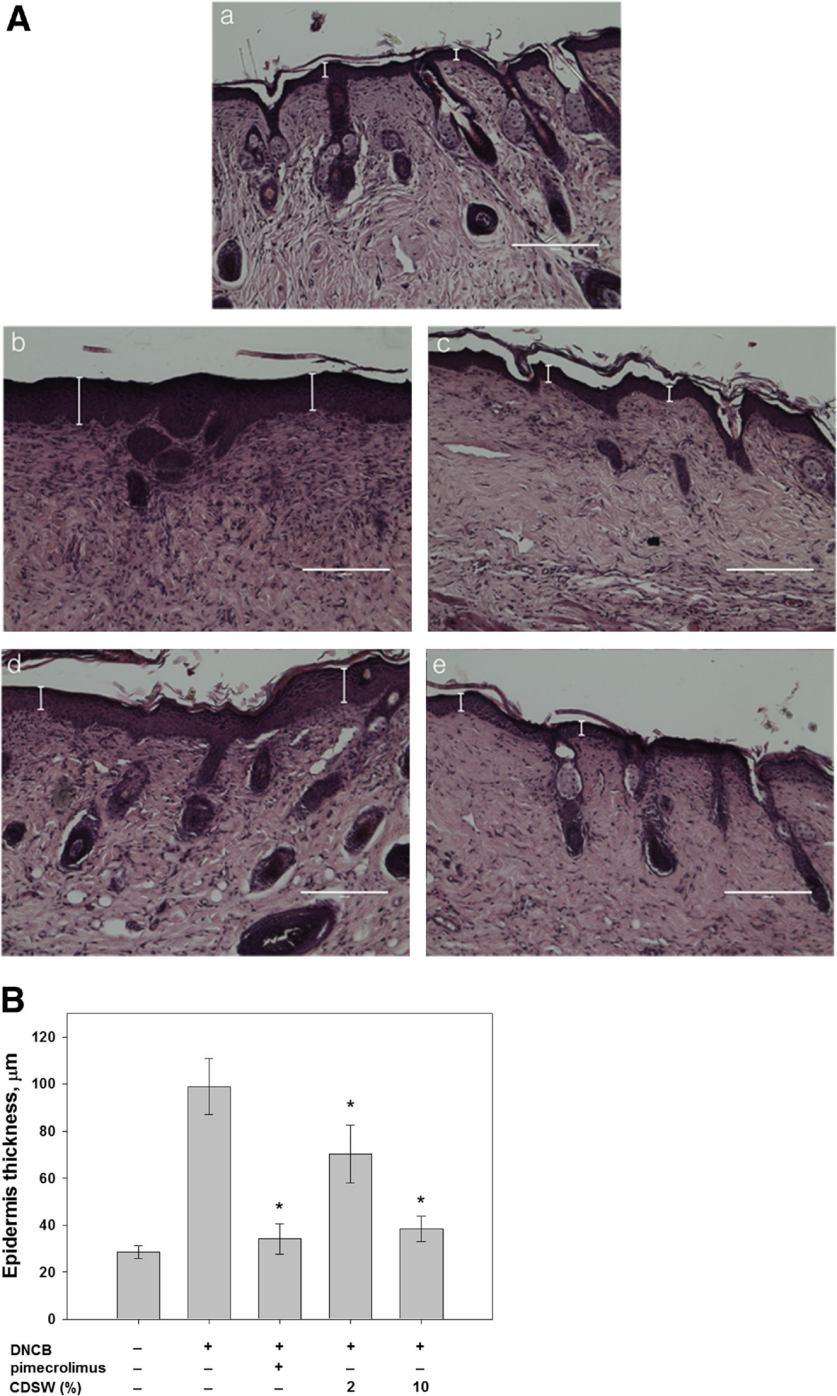
**Change in skin epidermal thickness elicited by the repeated topical application of DNCB in NC/Nga mice. A**) 8 μm-thick sections of DNCB-treated mice were fixed with formaldehyde and stained with hematoxylin and eosin; a. normal control, b. negative control (DNCB), c. positive control (DNCB + 1% pimecrolimus), d. 2% CDSW-treated (DNCB + 2% CDSW) e. 10% CDSW-treated (DNCB + 10% CDSW); (n = 10, magnification 200x, scale bars 200 μm). **B**) Epidermal thickness was measured by Micron (EVOS, v2.0) a digital imaging software (*p < 0.05).

**Figure 4:**
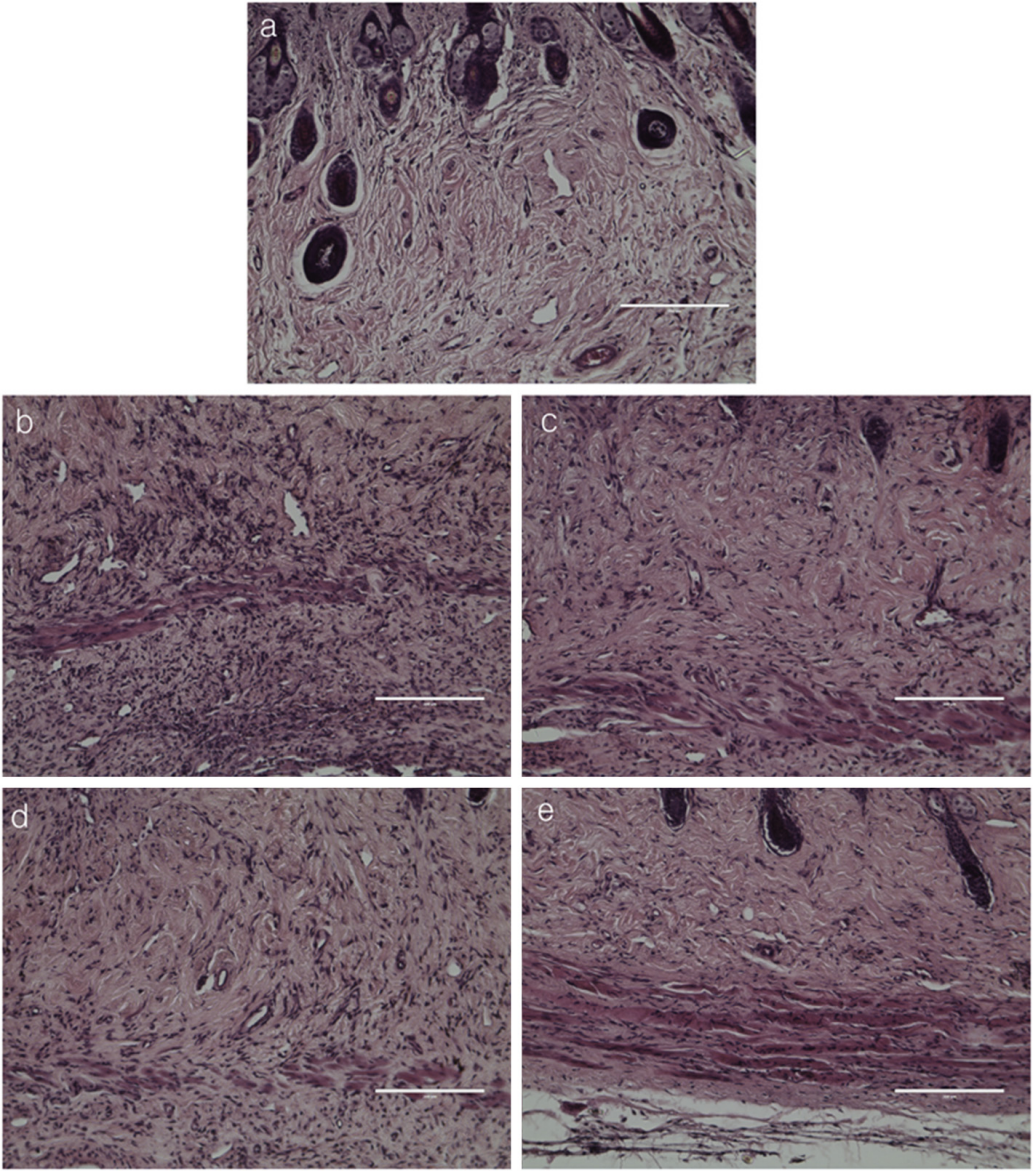
**Histopathology of skin lesions in DNCB-treated NC/Nga mice after treatment with DNCB.** Histopathological features of skin lesions were fixed with formaldehyde and stained with hematoxylin and eosin; **a.** normal control, **b.** negative control (DNCB), **c.** positive control (DNCB + 1% pimecrolimus), **d.** 2% CDSW-treated (DNCB + 2% CDSW) **e.** 10% CDSW-treated (DNCB + 10% CDSW); (n = 10, magnification 200x, scale bars 200 μm).

To further investigate the effect of CDSW on developing AD skin lesions, we used H&E staining to examine changes in epidermal thickness and reduced infiltration of inflammatory cells in the dermatitis. DNCB-treated mice had increased epidermal thickness that was restored to normal levels by treatment with 1% pimecrolimus or 10% CDSW (Figure 3). In addition, by means of histologic analysis, DNCB-elicited lesions showed increases in dermal edema and infiltration of inflammatory cells in the dermis when compared with the normal control group (Figure 4). The skin lesions of 1% pimecrolimus-treated group were prevented developing AD and were comparable to the normal control group. However, the 2% CDSW-treated mice showed many symptoms of AD while the 10% CDSW-treated showed much improvement. Thus, these data indicate that treatment of CDSW to AD skin lesions inhibited development of dermatitis induced by DNCB.

### Effect of CDSW on DNCB induced allergic response

**Figure 5:**
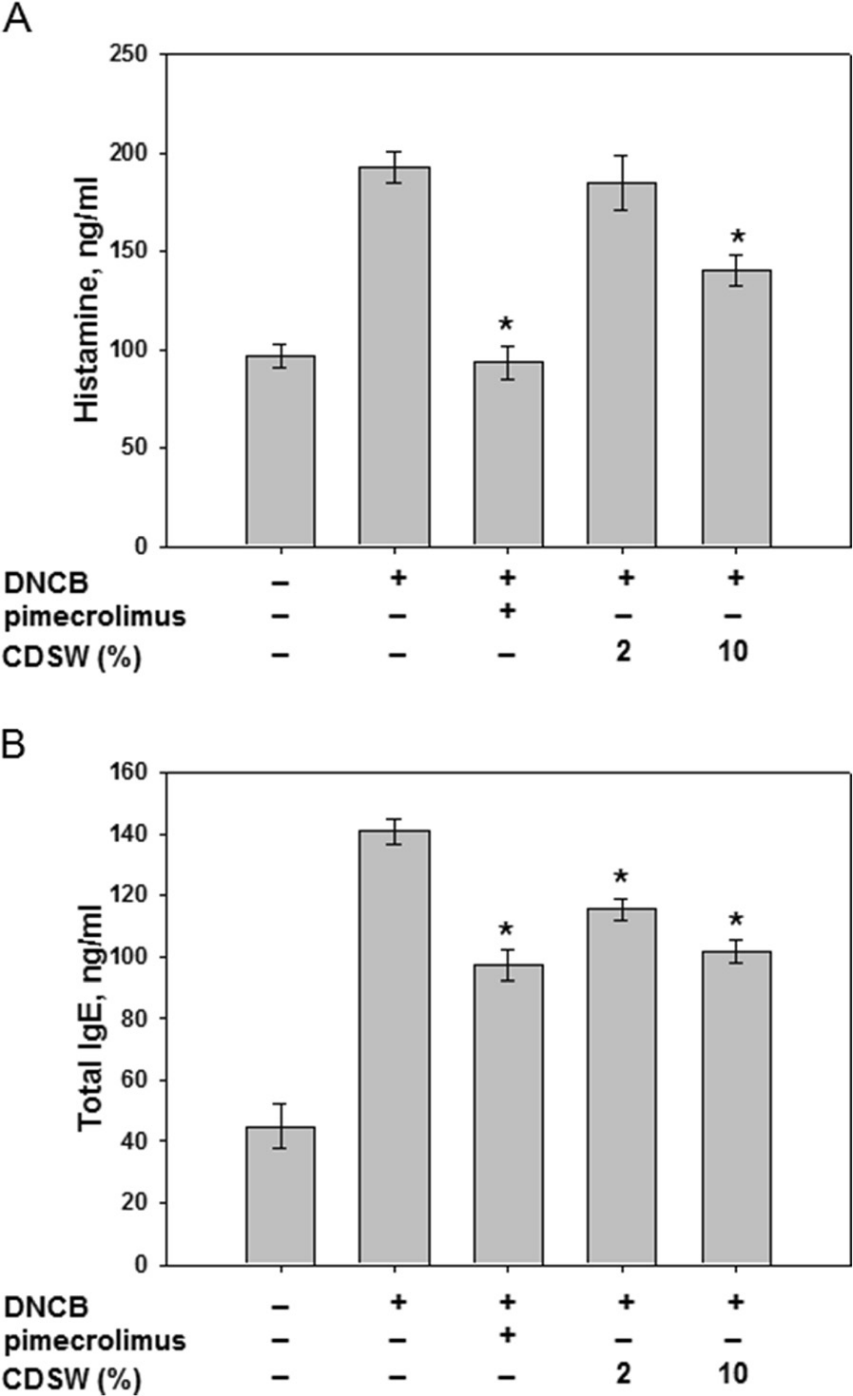
**CDSW inhibited IgE and histamine levels of DNCB-sensitized NC/Nga mice.** The serum prepared from heart in each group mice (n = 10). **A**) Histamine and **B**) IgE levels in serum were determined by ELISA as described in Materials and Methods. The data represent the mean ± S.D. of experiments performed in triplicate (*p < 0.05).

It was reported that the clinical severity of AD was associated with up-regulation of serum IgE and histamine levels. To access the effect of CDSW on allergic responses, the serum from each group of mice were collected and analysed. Repeated application of DNCB caused an elevation of total IgE levels in the serum and 10% CDSW treatment significantly reduced this effect (Figure 5). Also, we measured the release of histamine which is downstream of IgE. Histamine levels were increased significantly by DNCB treatment and the inhibitory effect of CDSW on DNCB-induced histamine release was observed (Figure 5). CDSW inhibited DNCB-induced total IgE levels and histamine release at concentration-dependent manner. These results indicate that CDSW lowers serum histamine and IgE levels in DNCB induced murine AD model.

**Figure 6:**
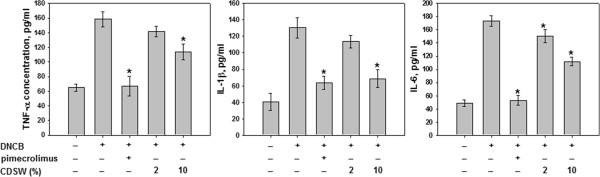
**Proinflammatory cytokine production by serum prepared from NC/Nga mice.** The serum prepared from hearts in each group of mice (n = 10). IL-1β, IL-6 and TNF-α levels in serum were determined by ELISA as described in Methods. The data represent the mean ± S.D. of experiments performed in triplicate (*p < 0.05).

The inhibition of pro-inflammatory cytokines including IL-1β, IL-6 and TNF-α is important characteristics of anti-inflammatory activities; therefore, we examined whether CDSW can inhibit expression of pro-cytokine production. Secretion of pro-inflammatory cytokines, TNF-α, IL-1β and IL-6, was increased markedly by DNCB treatmet but CDSW inhibited the production of cytokines (Figure 6).

T-cells have critical roles in the immune system and include two major classes; CD4+ cells are helper cells and CD8+ cells are suppressor cells [31]. CD4+ cells lead the attack against pathogenic infections, and CD8+ cells lead to ending immune response. We found that CDSW suppressed elevation of cutaneous inflammatory cells number (Figure 4). To verify the effect of CDSW on activation of inflammatory cells, we measured the ratio of CD4+/CD8+ by FACS (Table 2). The ratio of CD4+/CD8+ is 2.03 ± 0.362 in the normal control group and increased (4.96 ± 0.685) in the DNCB-treated group. However, 1% pimecrolimus ointment and 10% CDSW significantly reduced the CD4+/CD8+ ratio induced by DNCB (Table 2).

**Table 2:** The levels of T cells from spleen

**Group**	**CD4^+^, %**	**CD8^+^, %**	**CD4^+^/CD8^+^, %**
Normal control	3.45 ± 0.332^**a**^	1.62 ± 0.185	2.13 ± 0.153^**a′**^
DNCB	8.65 ± 0.455^**b**^	1.78 ± 0.267	4.96 ± 0.685^**b′**^
DNCB + 1% pimecrolimus	3.87 ± 0.619^**a**^	1.66 ± 0.273	2.41 ± 0.533^**a′**^
DNCB + 2% CDSW	8.15 ± 0.585^**b**^	1.76 ± 0.216	4.70 ± 0.627^**b′**^
DNCB + 10% CDSW	4.25 ± 0.420^**a**^	1.67 ± 0.213	2.58 ± 0.404^**a′**^

CD4+ and CD8+ cells were from spleen and were determined by FACS (fluorescence-activated cell sorter). Data are mean ± SEM of 10 mice. ^a-b, a′-b′^Values without the same superscript letter are significantly different at *P* < 0.05.

**Figure 7:**
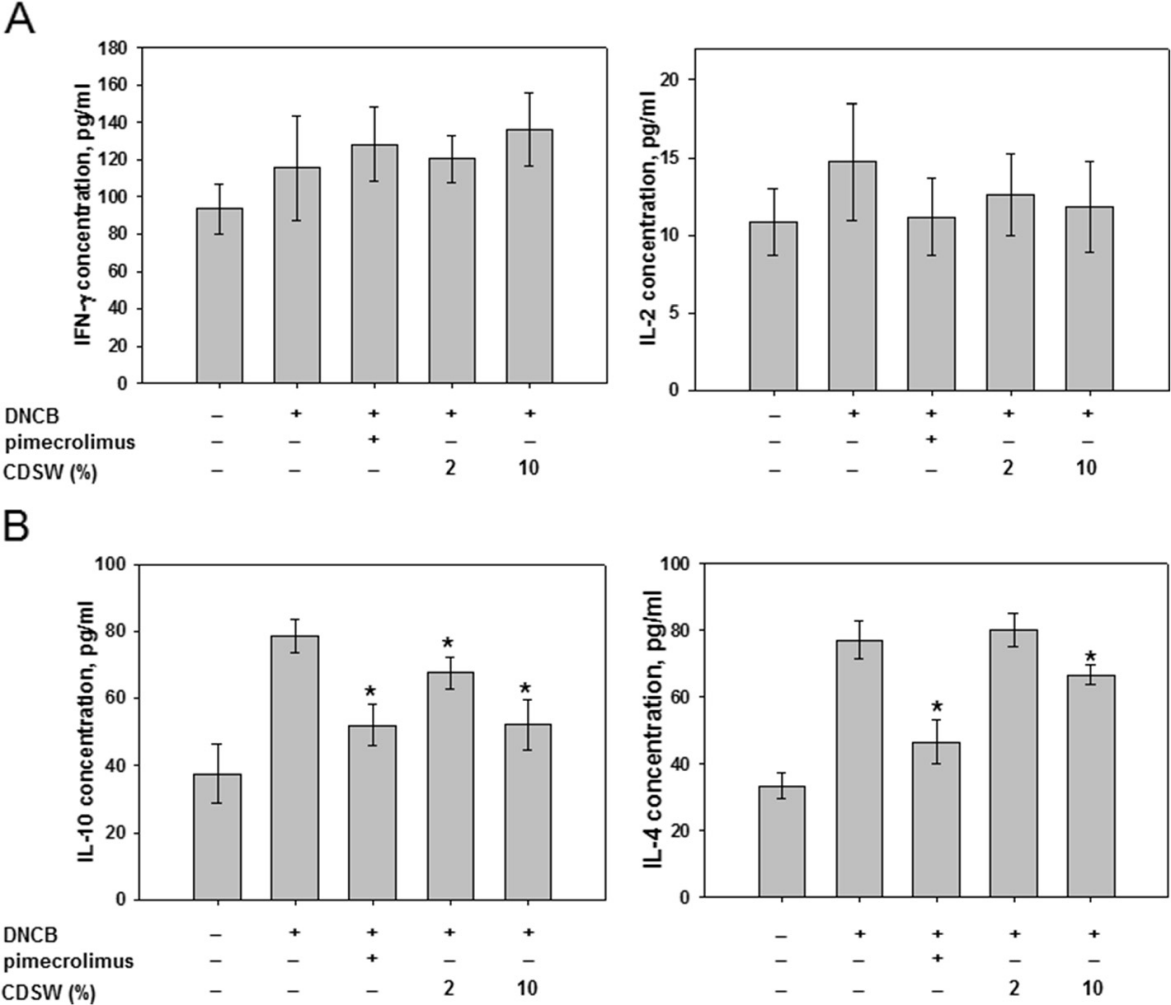
**Effect of CDSW on the levels of serum cytokine in DNCB-treated NC/Nga mice.** The serum prepared from hearts in each group of mice (n = 10). IL-2, IL-4, IL-10 and IFN-γ levels in serum were determined by ELISA as described in Methods. **A**) IL-2 and IFN-γ from Th1 lymphocytes and **B**) IL-4 and IL-10 from Th2 lymphocytes. The data represent the mean ± S.D. of experiments performed in triplicate (*p < 0.05).

We also determined the expression of Th cell development cytokines. IL-2 and IFN-γ, from Th1 cells did not show significant change after DNCB-treatment or application of CDSW (Figure 7A). Interestingly, IL-4 and IL-10 expression from Th2 cells were reduced significantly in the 10% CDSW-treated group (Figure 7B). These results were consistent with our macroscopic analysis and histological data. In these experiments, treatment with 10% CDSW suppressed the enhanced allergic responses induced by DNCB.

## Discussion

Atopic dermatitis is one of the many diseases induced by environmental elements as an antigen and the incidences of AD have sharply increased recently [5-9]. AD is a common chronic inflammatory skin disease, although fundamental etiology and therapy is still poorly understood [6-9]. Treatment with steroid-based drugs has been used for treating AD, but has encountered some problems [21].

It is well known that Dead Sea water has beneficial effects for treating skin diseases, including AD [32]. Recent studies reported that Dead Sea water has been used to treat psoriasis and atopic dry skin [32]. Dead Sea water is rich in some minerals compared to ocean water. Minerals, are essential nutrients for human, can trigger alterations on gene expression by initiating signaling events upstream of gene transactivation. Until the present, there are few studies for minerals and AD in human, however, the studies of mice reported that *Staphylococcus aureus* was increased on the skin of zinc-deficient mice before the development of AD-like eruptions, leading the authors to postulate that zinc may have an important role in the induction of dermatitis [33]. The deficiency of magnesium also induced AD-like skin lesions [34]. Because DSW also has enough minerals as well as Dead Sea water, in the present study, we demonstrate whether DSW also has an effect on amelioration of AD-like skin.

Before application of DSW to DNCB-elicited lesions, we screened the elemental composition of DSW and their concentrations (Table 1). There were some potential problems because DSW had high sodium concentration and salt-stress may induce inflammation [32]. Therefore, we examined CDSW, which was made by concentrating and desalinating DSW, and dilutions of CDSW were used. Although the concentration of the other elements in CDSW increased, the salt concentration was reduced (Table 1). Accordingly, we examined whether CDSW has an effect on AD induced by DNCB treatment in mice.

Although some symptoms of AD remained slightly, we have shown that repeated application of 10% CDSW improved the clinical severity score in DNCB-treated mice compared with a negative control. We scored five symptoms in skin lesions including itching, erythema, edema, excoriation/erosion and scaling/dryness to evaluate clinical skin severity. Among the treated animals, the 10% CDSW group had some edema, erosion and erythema, and did not differ dramatically from the 2% CDSW group. However, the itching was largely reduced by treatment with 10% CDSW. We also measured we measured TEWL and the moisture content in the epidermis and found that the 10% CDSW group had significantly improved skin barrier function and epidermis moisture. In addition, as demonstrated by histologic analysis, the 10% CDSW treatment reduced the infiltration of inflammatory cells, such as leukocytes and mast cells. These findings suggest that CDSW may restore skin barrier function.

Human AD disease is characterized by increased levels of Immunoglobulin E (IgE) in the blood [35]. IgE plays an important role in allergic responses and is especially associated with type-1 hypersensitivity. IgE is secreted from B cells by external antigens such as pollen and house dust mites [10,35]. Recent studies have reported that AD severity is related to an increase in total serum IgE levels [36,37]. Indeed, in our study, the clinical skin severity of DNCB-induced dermatitis was increased in accordance with up-regulation of total IgE levels in the serum. Mast cells are one class of inflammatory cells and the activation and degranulation of mast cells is tightly regulated by IgE. Total IgE affects mast cells and induces degranulation and triggers secretion of histamine and other inflammatory mediators [10,35,38]. Among the released inflammatory mediators, histamine is one of the most potent mediators [38]. In AD skin lesions, mast cells infiltrate the dermis and elicit inflammation by releasing histamine and other mediators. Infiltration of inflammatory cells in the dermis, induced by DNCB, was inhibited by 10% CDSW (Figure 4) and significantly reduced total IgE and histamine levels in the serum. These results indicate that CDSW lowers serum histamine and IgE levels in DNCB induced murine AD model.

It was believed that inflammatory cytokines contribute to inflammation in skin lesions in AD [15]. T-helper cells (CD4+), a type of white blood cell, play an important role in the immune system and have two subfamilies with distinct regulatory and influencing functions, based on their cytokines: Th1 lymphocyte and Th2 lymphocyte [39]. Th1 lymphocytes produce IFN-γ and IL-2, while Th2 lymphocytes produce IL-4 and IL-10. Excessive release of these cytokines is an important part of inflammation in AD. Among these, it was reported that Th2 cytokines regulate IgE synthesis. Treatment of DNCB promoted Th2 cell responses, such as IL-4 and IL-10 production. Overexpressed IL-4 promotes the development of AD [40]. In our experiments, production of IL-4 and IL-10 was reduced by 10% CDSW treatment, which led to reduced serum IgE levels. Treatment with 10% CDSW did not have any significant effect on Th1 responses. Taken together, our data show that application of CDSW has a beneficial effect for treating AD-like symptoms.

## Conclusions

In conclusion, our data show that application of CDSW to AD-like lesions significantly down-regulates Th2 cytokines, serum IgE levels and clinical severity scores. These observations lead us to conclude that topical application of CDSW to skin lesions alleviates at least some symptoms of atopic dermatitis. Also, it is possible that topical application of CDSW may prove to be an effective approach for preventing other allergic skin diseases.

## Competing interests

The authors declare that they have no competing interests.

## Authors’ contributions

JPB, YMK and JHS carried out the experimental portions of this research, such as animal experiments, ELISA assays, FACS analysis, evaluation of results, and writing of the manuscript. JPB, EHK and CHK supervised the work, wrote the manuscript and, designated the experimental work. All authors read and approved the final manuscript.

## Pre-publication history

The pre-publication history for this paper can be accessed here:

http://www.biomedcentral.com/1472-6882/12/108/prepub

## Supplementary Material

Additional file 1 **Figure S1.** The diagram of desalinization process and nanofiltration system. Click here for file

Additional file 2 **Figure S2.** The process of concentrated DSW from DSW. Click here for file

Additional file 3 **Figure S3.** Experimental schedule for the induction, treatment and evaluation of AD-like lesion in NC/Nga. Click here for file
