# Supplementary figures and images for: Application of concentrated deep sea water inhibits the development of atopic dermatitis-like skin lesions in NC/Nga mice
Source: BMC Complement Altern Med. 2012 Jul 26;12:108. doi: 10.1186/1472-6882-12-108 (PMC3517761; doi:10.1186/1472-6882-12-108)

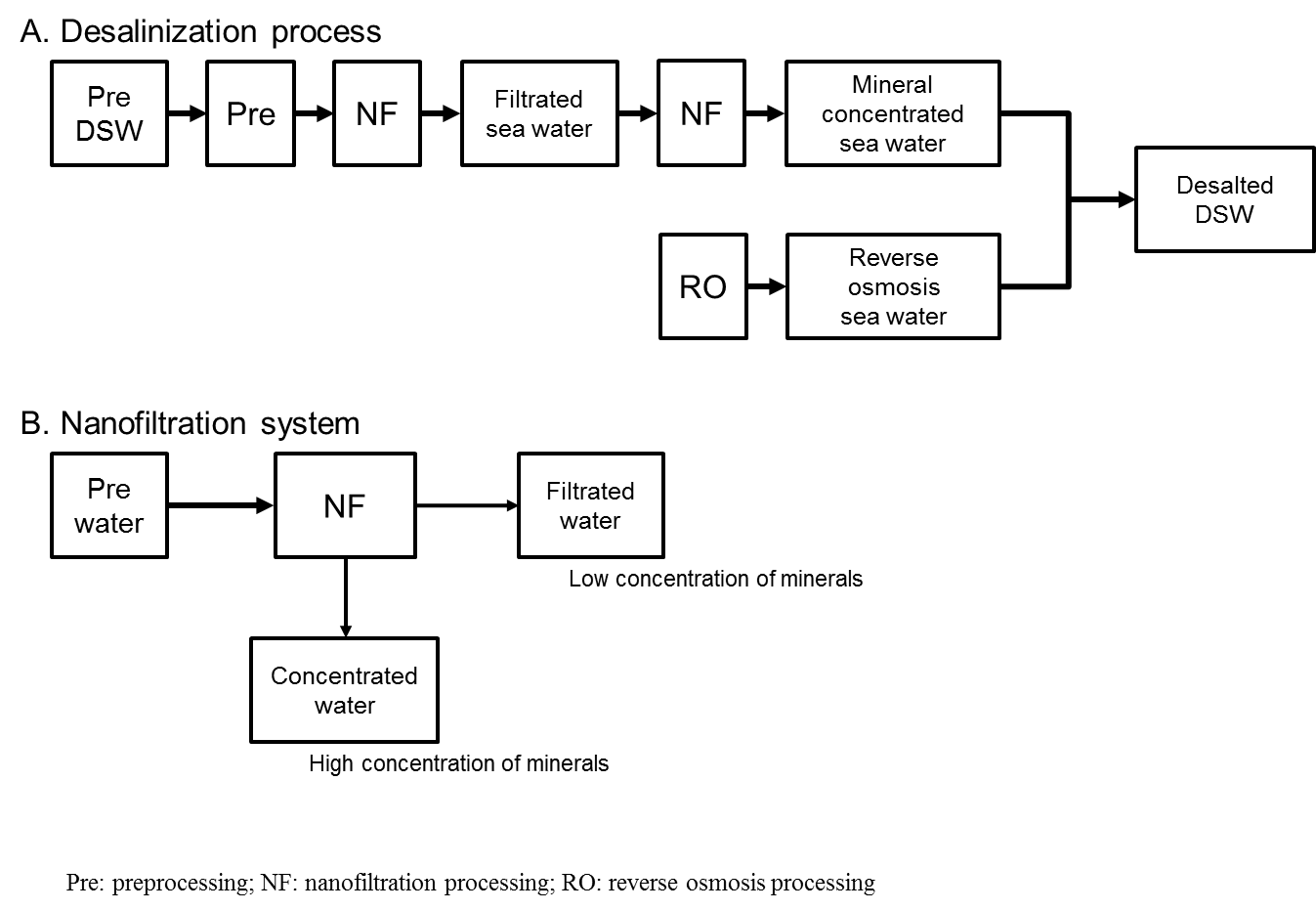

Supplement: Additional file 1 — Figure S1. The diagram of desalinization process and nanofiltration system. [file 1472-6882-12-108-S1.tiff]

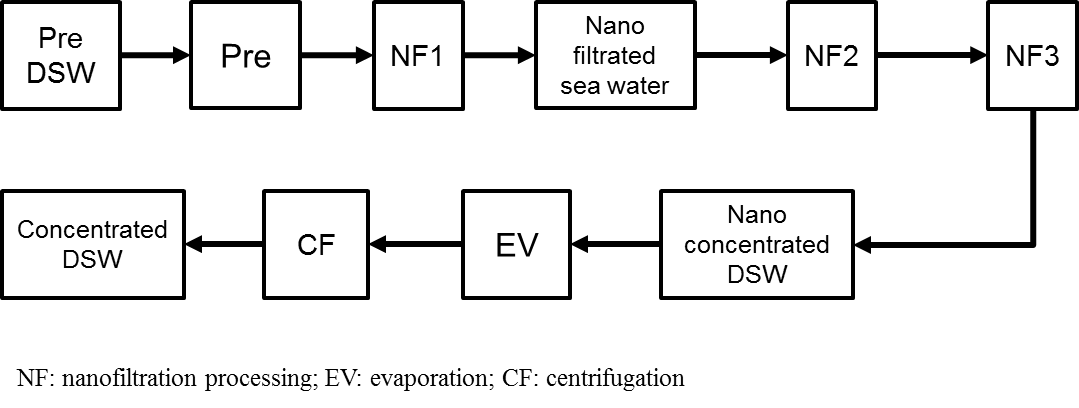

Supplement: Additional file 2 — Figure S2. The process of concentrated DSW from DSW. [file 1472-6882-12-108-S2.tiff]

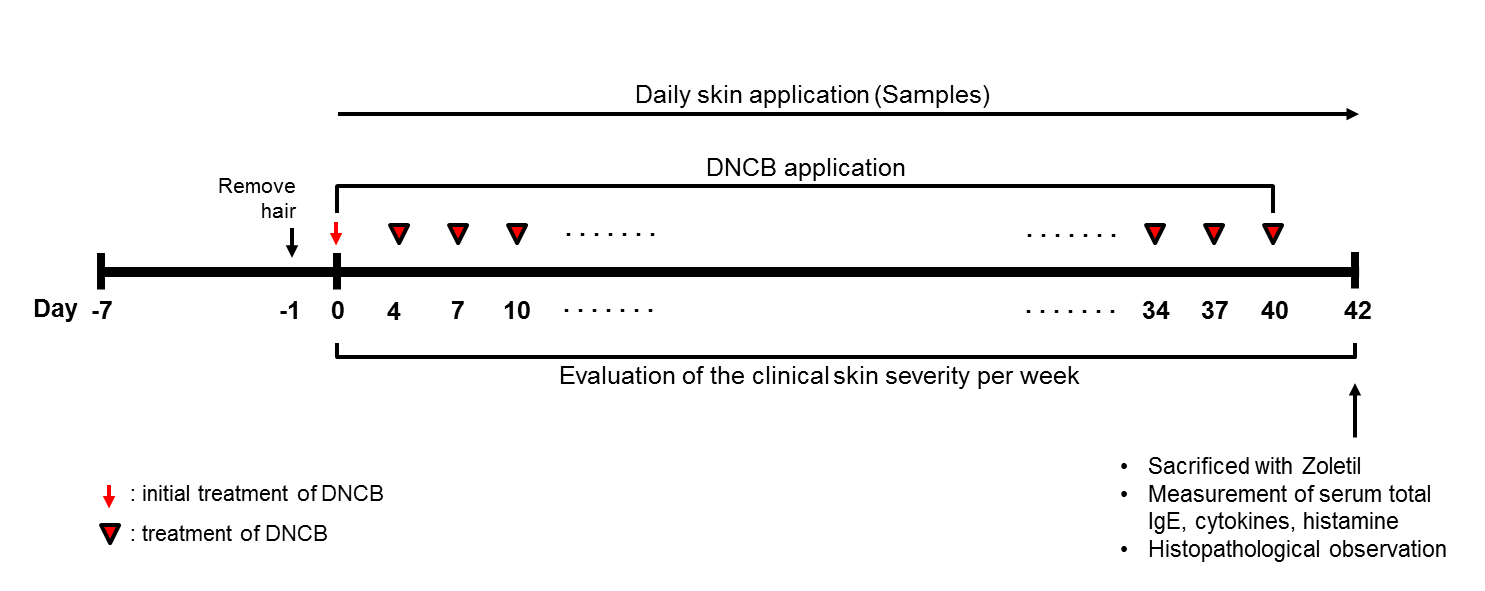

Supplement: Additional file 3 — Figure S3. Experimental schedule for the induction, treatment and evaluation of AD-like lesion in NC/Nga. [file 1472-6882-12-108-S3.tiff]
